# A Holistic Approach to the SMILE Mission and SMILE Public Engagement

**DOI:** 10.1007/s11214-025-01175-5

**Published:** 2025-06-11

**Authors:** Jennifer Alyson Carter, Steven Sembay, Simona Nitti, Maria-Theresia Walach, Steve Milan, Yasir Soobiah, Kjellmar Oksavik, Colin Forsyth, Matthew G. G. T. Taylor

**Affiliations:** 1 School of Physics and Astronomy, University of Leicester, University Road, Leicester, LE1 7RH UK; 2 Space and Planetary Physics Group, Physics Department, Lancaster University, Lancaster, LA1 4YB UK; 3 Department of Physics and Technology, University of Bergen, Bergen, Norway; 4 Arctic Geophysics, University Centre in Svalbard, Longyearbyen, Norway; 5 Mullard Space Science Laboratory, UCL, Dorking, RH5 6NT UK; 6 ESTEC, European Space Agency, Noordwijk, The Netherlands

**Keywords:** SMILE, Magnetopause, Ionosphere, Solar wind charge exchange X-ray emission, Solar-terrestrial, Ultraviolet aurora

## Abstract

Here we consider initial steps of how upcoming data from the SMILE Soft X-ray Imager and Ultraviolet Imager may be combined with additional data sources to provide a more holistic view of the coupled magnetosphere-ionosphere system. The Ground-based and Additional Science Working Group aims to embed SMILE in a multi-scale and holistic view of the Earth’s magnetosphere by exploring coordination of ground-based and other spacecraft’s data with SMILE. This working group is one of four working groups within the SMILE Science Working Team who are tasked with preparing all aspects of the mission. Adequate preparation is essential to optimise the tools, multiple instrument campaigns and procedures to allow the maximum science return from SMILE in the context of the entire available range of temporal and spatial scales in the terrestrial system. SMILE instruments will not work in isolation from each other, nor from other spacecraft or ground-based experiments. Synergies with other missions and ground-based experimentation will be fundamental for full science exploitation of the data. In this paper, we expand on the previous publications by the Ground-Based and Additional Science working group, by exploring the possibilities of using a two-way approach to deriving scientific results from SMILE, using a small isolated substorm as a case study. We use knowledge of the contemporaneous solar wind conditions during the substorm to simulate SMILE Soft X-ray Imager data. We also use observed ultraviolet auroral emissions and field-aligned current data as measured in the high-latitude polar regions to act as either a proxy for the SMILE Ultraviolet Imager, or an alternative source of information for the open-closed field line boundary. The observational data is used to constrain the minimisation of the two-dimensional X-ray images, leading to an improvement in the derived shape of the flank magnetopause position. We also comment on mission’s possibilities to inspire the public through various engagement programmes, and current activities to involve diverse communities in the preparations and science exploitation of SMILE.

## Introduction

The Solar wind Magnetosphere Ionosphere Link Explorer (SMILE) mission is due to launch in 2026 to a high-inclination, high-altitude orbit with an apogee of 20 $R_{E}$ which offers long science duty cycles of approximately two days (Branduardi-Raymont et al. 2018, and papers in this collection). Using its two imaging cameras with offset field of view, SMILE will observe large-scale dynamics of the dayside magnetosheath, while simultaneously observing the response of the ionosphere. The Soft X-ray Imaging (SXI, Sembay et al. 2024) camera will image the dayside magnetosheath, using a large field of view telescope of 25.5 degrees by 15.5 degrees. The Ultraviolet Imager (UVI) will observe the Northern Hemisphere high-latitude ionosphere through emissions in the range 160 nm, to 180 nm, encompassing part of the $\mathrm{N_{2}}$ Lyman Birge Hopfield band. The imaging instruments are accompanied by two in situ instruments; the Light Ion Analyser (LIA) and the magnetometer (MAG). By combining all the SMILE instruments with the wealth of available data across the globe, will we be able to obtain a truly holistic picture of solar-terrestrial dynamics.

Due to the driving ambitions of the SMILE Science Working Team to enable joint analysis and coordinated science campaigns between SMILE and ground-based or other space-based experimentation, the Ground Based and Additional Science (GBAS) working group was formed (Carter et al. 2024). This working group is preparing tools and infrastructure to allow timely science returns from experiments that will operate simultaneously to SMILE, and is exploring techniques and procedures that could be optimized for the solar-terrestrial community well before launch. This includes a dedicated Northern Hemisphere winter campaign, under dark conditions and during favourable sky conditions around new Moon that will allow for dedicated multi-facility experiments to investigate particular science goals (Walach et al. 2024). Tools include the SMILE Data Fusion Facility (Carter et al. 2024), to plan coordinated observations in both hemispheres, using the currently available SMILE spacecraft ephemeris. These plans are fuelled by an increased realisation that symbiotic relationships between spacecraft, potentially at different orbits and carrying different payloads and with, at least initially, apparently disparate science targets, provide an efficient and holistic perspective on the most pressing science questions (https://eo4society.esa.int/wp-content/uploads/2021/11/swarm_report_211112.pdf, McGranaghan et al. 2023a,b; Kepko et al. 2024) SMILE offers an opportunity for the upstream results as derived from SXI data to inform investigations throughout the magnetosphere but also for the reverse to be true, so that phenomena or states in the inner magnetosphere may inform the interpretation of those upstream.

The SXI Consortium is obliged to provide calibrated photon lists (see Sembay et al. 2025, this issue), nominally at 10-minute intervals. From these calibrated photon lists, a user may select photons to construct an image, create a time series, or a spectrum, in an energy band of interest over an integration time of interest, with the option to concatenate lists as required. This approach gives a user complete flexibility on how they process the data. An extremely important data product for the community, however, will be the derivation of the magnetopause and cusp boundaries. A user has free reign to derive these boundaries themselves, following their own method or those being developed by the SMILE Modelling Working Group (Wang and Sun 2022) using the calibrated photon lists. Nevertheless, canonical boundary positions (Level 4) produced by the SXI team will be provided for use by the community, even though this requires implied assumptions on integration times and the method applied. These Level 4 products will be of wide use as quick looks and for science, when the assumptions are sufficient for the questions to be pursued.

In this paper, we consider the SMILE imaging instruments working together, rather than in isolation, and in the context of additional information provided by either ground- or space-based experimentation. This paper considers a set of techniques that may be used when combining SXI and UVI data, and how the instruments working together may be mutually supportive, along with complementary ground or space-based data from other experiments. For this, we used the case of an isolated substorm with supporting space-based data sets that are currently available. We examine simulated SXI images, the SXI-derived magnetopause boundaries from these simulated images, and the implications of the boundaries as field line traced to the ionosphere. We used previous observations of auroral emissions using spacecraft auroral imagers as a proxy for UVI, from which we estimate the open-closed field line boundary (OCB) that can be traced outwards from the ionosphere to the magnetopause. In practice, the UVI observations can be used in combination with observations from either operational ground-based or space-based imagers. The OCB is significant because it is used to determine changes in the open magnetic flux of the polar cap as it responds to energy input following magnetic reconnection at the dayside magnetopause, for example (Milan et al. 2003), balanced by any closure of magnetic flux in the magnetospheric tail (Walach et al. 2017; Mooney et al. 2020). We test whether the estimation from this outward field-line tracing is able to constrain the boundary detection methods employed when determining the magnetopause boundary with SXI, and so provide a demonstration of one method that might be used once observation data become available post-launch. We also consider SMILE data products and the mission for public engagement and outreach purposes through a separate working group within the SMILE Science Working Team, where there is considerable overlap in membership with the GBAS working group. These members also have a wealth of experience in bringing solar-terrestrial science to the general public, and not restricted by a particular mission. We describe some of the intentions of the working group for interactive programmes, events, and materials.

## Data and Simulations

**Fig. 1:**
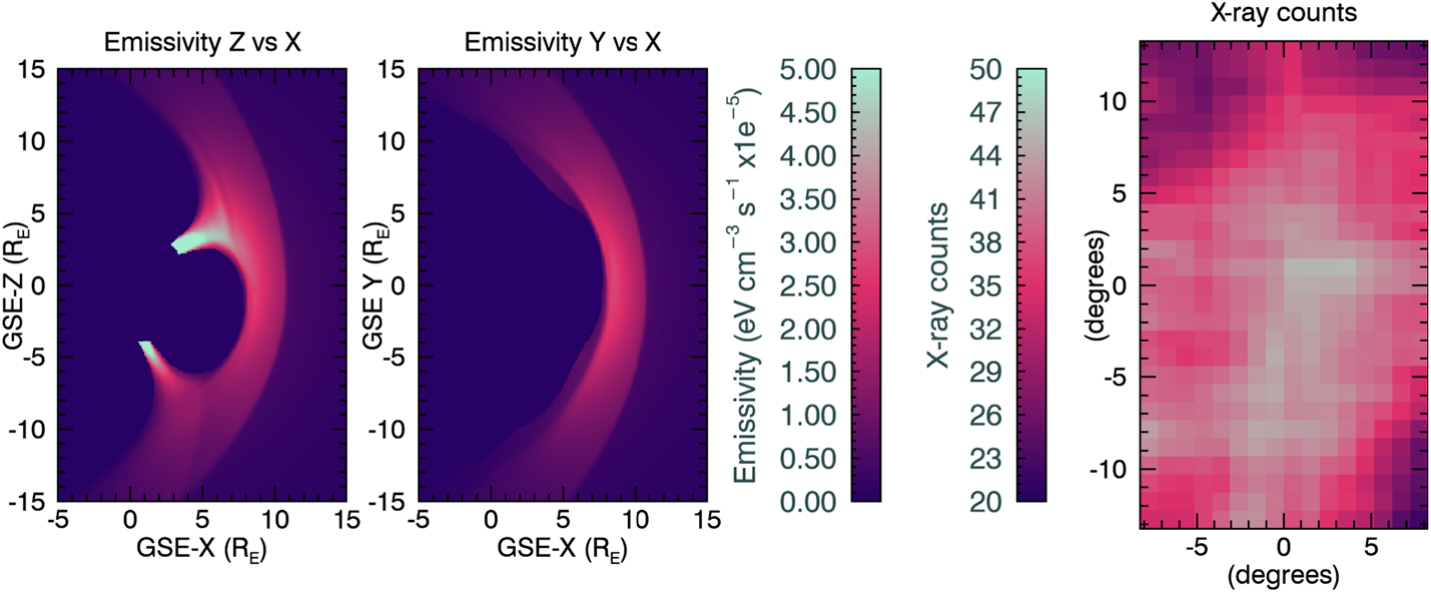
Example X-ray emissivity slices in the XZ and XY planes, plus SXI simulated counts, for one time stamp, applicable to conditions during the substorm on 28 May 2010. The SMILE spacecraft was placed at a GSE position of 6.05 $\mathrm{R_{E}}$, 7.23 $\mathrm{R_{E}}$, 17.39 $\mathrm{R_{E}}$ in the X, Y, and Z directions respectively, and was aimed at the dayside magnetosphere. This image has been smoothed using a boxcar average with a width of 5 pixels. The Sun is to the right in the positive X direction

SXI simulations require inputs of three-dimensional cubes of X-ray emission from the Earth’s magnetosheath resulting from the solar wind charge exchange (SWCX) process, so that two-dimensional images of integrated lines of sight from a particular SMILE spacecraft vantage point can be obtained (e.g. Sibeck et al. 2018). Figure 1 shows images of simulated X-ray emissivities in the XZ and XY planes in the left and middle panels. These pure foreground emissions are later convolved through the telescope optics and instrument response to produce simulated X-ray count-rate images, as shown in the right-hand panel of the figure, with assumptions for instrumental noise and astrophysical background. The SXI instrument simulator is described in Sect. 2.3. The emissivity cubes are constructed by assuming an underlying exospheric hydrogen density distribution, which is then multiplied by distributions of solar wind plasma in and around the Earth’s magnetosphere, assuming an efficiency of the charge-exchange process that results in near-Earth X-ray emissions. These cubes of simulated X-ray SWCX emission have been widely used in the preparations for SMILE so far, and during precursor studies like (e.g. Sun et al. 2019). In the majority of simulations run so far in preparation for the SMILE mission, we have used magnetohydrodynamic (MHD) code to estimate the densities and speeds of solar wind protons as proxies for the solar wind heavy ions that undergo charge exchange. In this paper, we use MHD simulations from the Community Coordinated Modeling Center. Masking of apparent X-ray emissions from the inner magnetosphere plasma is required to avoid erroneous contributions to the line-of-sight integrated signal. For the simulations presented here, we use a method simpler than that of Samsonov et al. (2022) to remove inner magnetosphere emissions by excluding areas of low density. Our mask is constructed so that at each time step, a density cube is normalised between the minimum and maximum densities so that they run between unit-less values from 0 and 255, i.e. grey scaling. Any mask position with a value on this scale below 25 is excluded from contributing to the emissivity calculations.

### Solar Wind and Geomagnetic Conditions During Substorm on 28 May 2010

**Fig. 2:**
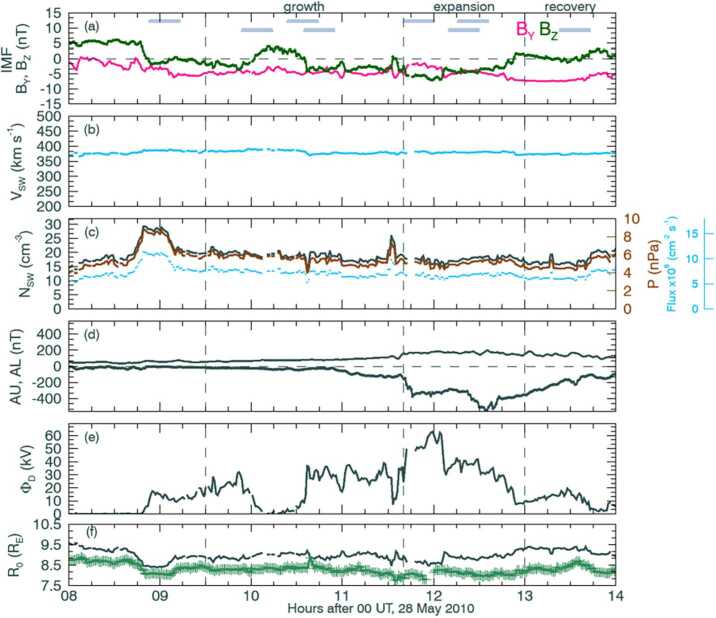
IMF, solar wind, and geomagnetic conditions for the case study. Panels (a-f) show; IMF $\mathrm{B_{Y}}$ and $\mathrm{B_{Z}}$ components in pink and green, respectively, solar wind speed, solar wind density and pressure, AU and AL auroral indices, the dayside reconnection rate, and the subsolar magnetopause position as estimated from the Shue and Song (2002) model in grey and as estimated from three-dimensional cubes of X-ray emissivity, marked as green crosses. Vertical dash lines mark out the substorm growth, expansion, and driven phases, as identified by Milan et al. (2021). The pale blue horizontal bars in panel (a) mark the duration of the DMSP/SSUSI Northern Hemisphere polar cap passes

The MHD code requires knowledge of the solar wind and interplanetary magnetic field (IMF), whose data we obtained through OMNI (King and Papitashvili 2005). Our case study was based on data from 28 May 2010. The IMF, solar wind, and geomagnetic conditions for this case are shown in Fig. 2. This period was chosen to represent a period of dayside reconnection under southward IMF, without evidence for nightside reconnection within contemporaneous auroral emissions and field aligned current data (Fig. 1 of Milan et al. 2021). This period represents a growth phase of a substorm during a southward turning of the IMF, as shown by the green $B_{Z}$ trace in panel (a) of the figure, during which the auroral oval will expand to lower magnetic latitudes as open magnetic flux is added to the lobes. The growth phase is preceded by steady speed solar wind, as shown in panel (b), but with a period of higher than average solar wind density and pressure, as shown in panel (c). The period is truncated by the onset of a substorm expansion phase shortly before 12 hrs UT. This is shown in the figure in the period defined between the second and third vertical lines, which is accompanied by a bay in the AL auroral index in the lower trace of panel (d), which later begins to return towards zero in the recovery phase. The dayside reconnection rate is calculated in (e), using the formula of Milan et al. (2012), and this rate is significantly enhanced during the expansion phase of the substorm. The Shue and Song (2002) model subsolar magnetopause location, which is dependent on the IMF $B_{Z}$ component and the dynamic pressure, is plotted in panel (f), along with the estimated subsolar position as implied by MHD-derived three-dimensional cubes of X-ray emissions in the magnetosheath, as shown by the green crosses. The SXI simulator count rate maps and the derivation of the magnetopause are described in Sect. 2.3.

**Fig. 3:**
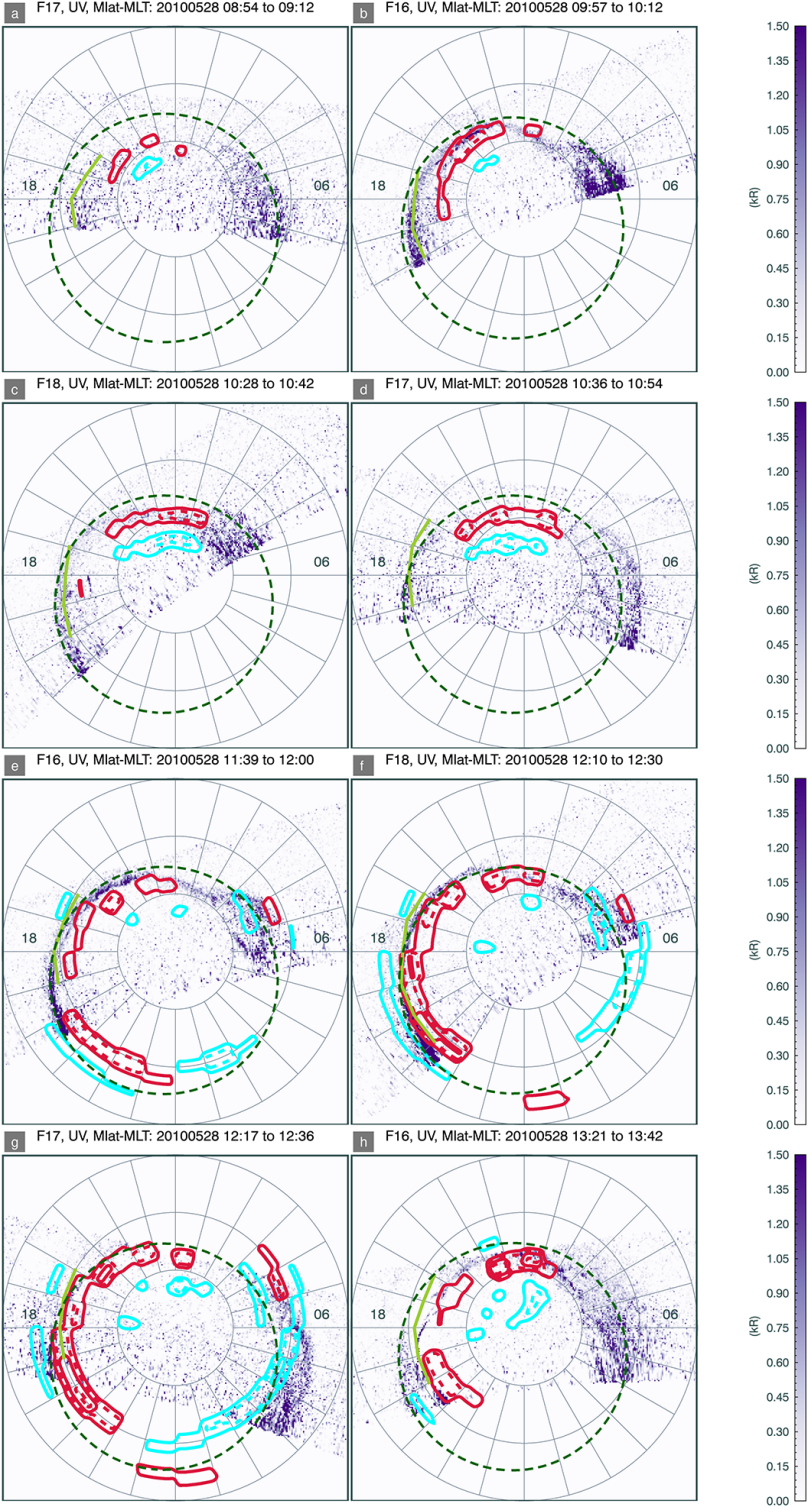
MLT-magnetic-latitude projections of DMSP/SSUSI LBH ultraviolet auroral emissions in Northern Hemisphere polar cap for the case study on 28 May 2010. Noon is to the top and dusk to the left. Contours of upwards and downwards FACs are plotted in red and blue, respectively at intervals of 0.25 $\mu A m^{-2}$ from absolute magnitudes of 0.5 $\mu A m^{-2}$ and higher. Estimated segments of the OCB in limited MLT sections of the afternoon or dusk flank are marked in lime green from the observational data. The dark green dashed circle marks the SXI-implied OCB, using a fixed flaring exponent, drawn at all MLTs by field-line tracing from the flank equatorial magnetopause up to the ionosphere and extending this to a circle, and is described in Sect. 2.3

**Fig. 4:**
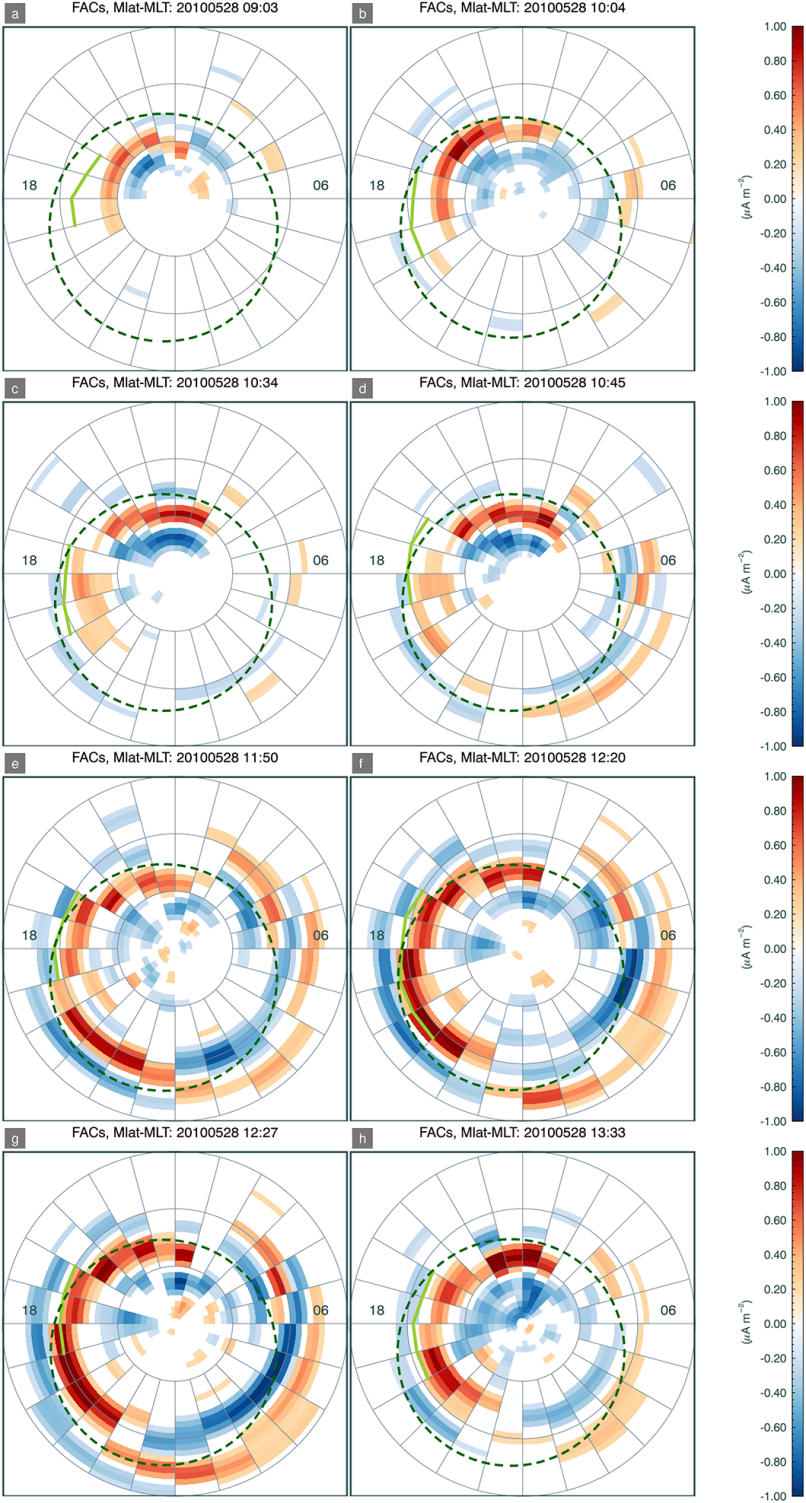
MLT-magnetic-latitude projections of AMPERE-derived field-aligned currents in the Northern Hemisphere polar cap for the case study on 28 May 2010. In the same format as Fig. 3

In Figs. 3 and 4 we plot a series of magnetic local time (MLT)/magnetic latitude projections of firstly auroral emissions, and secondly field-aligned currents during the substorm growth phase and into the beginning of the expansion phase. The Sun is towards the top of each image, and dusk to the left. Concentric rings are marked at intervals of 10 degrees magnetic latitude. Auroral emissions are obtained from the Special Sensor Ultraviolet Spectrographic Imager (SSUSI) (Paxton and Anderson 1992; Paxton and Zhang 2016) on board the Defense Meteorological Satellite Programme (DMSP) spacecraft from the satellites F16, F17, or F18. SSUSI has five spectral bands, but here we have plotted emissions in the Lyman Birge Hopfield (LBH) long band, representing $\mathrm{N_{2}}$ emissions in the band 165-180 nm, as this is close to the SMILE UVI instrument that will be sensitive to a combined LBH band from approximately 150 nm to 180 nm. SSUSI images are made up of cross-track scans as the spacecraft traverses the polar cap at approximately 800 km, taking about 20 min to do so. SMILE UVI will, in contrast to SSUSI, produce global images of electron-induced auroral emissions at approximately 1 minute cadence, and we are currently severely limited by the lack of a global auroral imager in operation. Field aligned current (FAC) densities at ionosphere altitudes have been obtained from the Active Magnetosphere and Planetary Electrodynamics Response Experiment (AMPERE) (Anderson et al. 2000; Waters et al. 2001), and are shown as either contours on the SSUSI images or separate images of FAC densities. Although the AMPERE data is available at 2 min cadence, each plot of FACs has been selected at a UT equivalent to the approximate midway point of the SSUSI scan.

### Determining the OCB from Ionospheric Data

The images of Fig. 3 and Fig. 4 allow an estimation of the OCB. The exact position of the OCB is a matter of considerable debate, and proxies for this boundary depend on a variety of measurements (e.g. Oksavik et al. 2000; Burrell et al. 2020; Carter et al. 2016). In situ monitors of particle precipitation note the OCB to lie poleward of the boundary as determined by precipitating proton-induced auroral emissions (Hubert et al. 2010). Ground-based observations of red-line auroral emissions place the dayside OCB equatorward of the cusp aurora since these emissions are produced by soft particle precipitation of magnetosheath origin (Johnsen and Lorentzen 2012), whereas proxies using auroral emissions from space-based instruments take the boundary to be poleward of the aurora, due to the assumptions that these emissions result from higher energy magnetospheric particle precipitations that is expected on closed, rather than open field lines (Longden et al. 2010). Here, we assume this OCB to lie on the poleward edge of the main auroral oval in the dayside polar cap, when this can be determined.

At certain times, it is possible to ascertain a strong UV emissions signal in the dayside polar region and hence a proxy for the OCB. At other times, the UV emissions are weak. When this occurs, the FAC densities may be used as an alternative proxy for the OCB if the region 1 currents are discernible. We assume that this FAC-boundary occurs between the region 1 and region 2 FACs, i.e. equatorward of the region 1 currents which are upwards in the dusk flanks (Milan 2013). This FAC-derived boundary may sit a few degrees equatorward of the OCB (Burrell et al. 2020), however, Korth et al. (2014) found that region 1 and discrete auroral electron-driven UV emissions are co-located, particularly in the dusk sector. On each plot, where possible to determine an approximate boundary position in the afternoon or evening sectors we plot an estimation of this boundary, judged from both the auroral and FAC data sets, on a panel. Note, that we have been careful in this case to avoid areas of low-intensity commonly-named auroral web emission, for example in the ≤6 hr to 8 hr MLT sector in panels a-c of Fig. 3, and also to avoid clear incursions into the polar cap from phenomena such as possible bending arcs (Carter et al. 2015) in the dayside pre- or post-noon quadrants, for example in the afternoon sector at an MLT of 14 hr to 15 hr in Fig. 3 panels (b), (d), (e), and (f), or possibly transpolar arcs incurring from the nightside, for example in the post dusk sector of Fig: 3 panel (h).

**Fig. 5:**
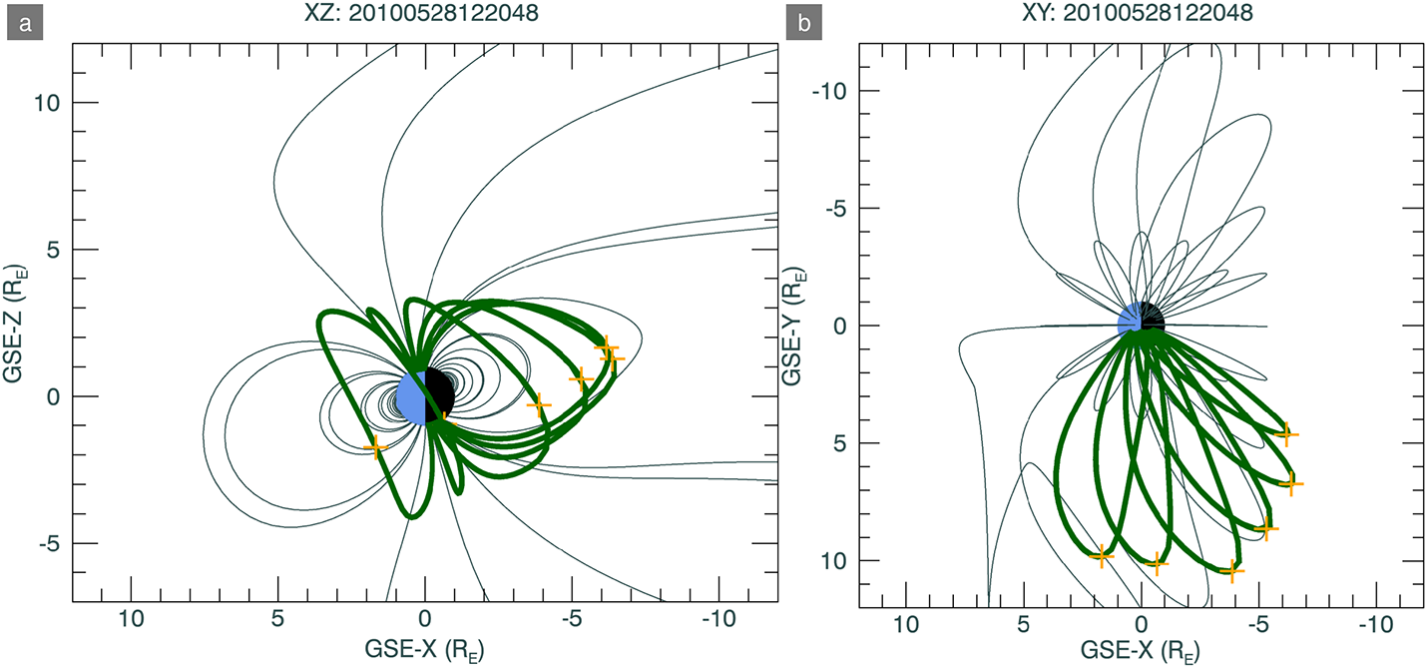
Example magnetic field model and field-line traced positions from the DMSP/SSUSI and AMPERE-derived positions of the OCB outwards to the flanks of the magnetopause for one time step in (a) the XZ plane, (b) the XY plane, with grey lines showing selected magnetic field lines of the magnetosphere, and those in green trace those selected from the ionosphere out to the magnetosphere flanks, where the equatorial positions are marked with orange crosses. The Sun is to the left

From the estimations of the OCB, as shown in Fig. 3, we trace the OCB outwards from the polar cap to the ecliptic flank of the magnetopause to give a set of magnetopause positions in the GSM Y-direction. Each flank position has an associated error. In Fig. 5 we plot an example of this field-line tracing of the OCB MLT/magnetic latitude positions in the ionosphere to the equatorial magnetopause, in both the XZ plane (a) and (b) XY plane. Using orange plus symbols, we mark the projection of those footprints traced to the magnetopause flanks which will later be used to contribute to the magnetopause fitting. To apply the field line tracing we used the IDL software Dynamic Link Module files that wrap the GEOPACK field-line tracing routines (see the section on Declarations), using the Tsyganenko T96 model (Tsyganenko 2013) of the Earth’s magnetic field with an IGRF internal geomagnetic field. The field line tracing is IMF and solar wind dependent, so we used the time stamps as shown in the FAC panels at approximately the mid UT of the DMSP/SSUSI polar cap crossing to select the relevant solar wind dynamic pressure, SYM-H geomagnetic index of ring current activity, plus the IMF $\mathrm{B_{Y}}$ and $\mathrm{B_{Z}}$ at each time, for each DMSP/SSUSI image used, as summarised in Table 1 and obtained from OMNI. We assumed an error in our determined OCB boundary of 1 degrees magnetic latitude. This is reasonable approximation on the error given the ∼100 km resolution of UVI.

**Table 1:** Auroral emission snapshots with singular solar wind and geomagnetic conditions, and traced limits to the subsolar magnetopause, for 28 May 2010. We list the DMSP satellite, time stamp of the mid UT for the DMSP polar cap crossing, solar wind dynamic pressure, SYM-H geomagnetic index, IMF $\mathrm{B_{Y}}$ and $\mathrm{B_{Z}}$ used for field-line tracing, the flaring exponent in the equatorial plane, $a_{y}$, and the implied distance to the subsolar magnetopause, $\mathrm{R_{MP_{0}}}$, as inferred through field-line tracing as described in the text, and Shue and Song (2002)

DMSP	Time	Inputs			Implied magnetopause		
		Dynamic	SYM-H	IMF	$a_{y}$	$\mathrm{R}_{\mathrm{MP}_{0}}$	$\mathrm{R}_{\mathrm{MP}_{0}, \mathrm{Shue}}$
		pressure		$\mathrm{B}_{\mathrm{Y}}$, $\mathrm{B}_{\mathrm{Z}}$			
	HH:MM	(nPa)	(nT)	(nT)		($\mathrm{R}_{\mathrm{E}}$)	($\mathrm{R}_{\mathrm{E}}$)
F17	09:04	6.90	31	−3.28, −1.42	-	-	8.44
F16	10:04	4.74	20	−5.10, 1.18	0.27 ± 0.35	8.70 ± 3.20	9.03
F18	10:34	4.61	14	−5.22, −0.02	0.29 ± 0.34	8.88 ± 3.22	9.03
F17	10:45	4.38	13	−4.48, −2.77	0.23 ± 0.23	9.06 ± 1.85	8.95
F16	11:50	4.25	9	−2.09, −5.55	0.12 ± 0.25	9.75 ± 2.12	8.67
F18	12:20	4.27	7	−3.74, −4.55	0.06 ± 0.15	9.81 ± 1.49	8.80
F17	12:26	4.37	8	−4.17, −4.19	0.33 ± 0.20	8.36 ± 1.55	8.82
F16	13:34	3.57	7	−7.11, 0.19	0.23 ± 0.22	10.06 ± 2.06	9.40

### SXI Simulations

We simulate SXI observations for the case on 28 May 2010, as described above and in the SXI paper, Sembay et al., 2025, found in this issue. We assume an orbit from the currently available estimated SMILE spacecraft ephemeris. The spacecraft position is moved from its assumed starting position of (4.87, 6.29, 18.09) in the geocentric solar ecliptic (GSE) frame, with a pointing direction that follows the spacecraft/instrument pointing law that maintains a fixed angle of around 20.25 degrees between the line-of-sight (LOS) to the edge of the Earth limb and the LOS to the edge of the SXI field of view. For the first pointing the centre of the field of view points towards a GSE position of (7.84, 0.00, 0.00). Each simulation is 20 minutes apart and the spacecraft position and pointing direction moved appropriately, although, as the chosen orbital positions are near apogee the relative change in the spacecraft position is small on this timescale (approximately 0.3% change in altitude relative to apogee). The SXI simulator contains a full background model including X-ray and particle induced components. The dominant source of background within the SXI primary science band (around 0.2 to 1.1 keV) is the astrophysical soft X-ray background from diffuse Galactic emission. We employ data from the ROSAT-PSPC all-sky survey (Snowden et al. 1995) to derive the estimated SXI background for this component using the relative response of both instruments to convert the observed PSPC count rates to SXI count rates. The general output of the simulator given a 3-D X-ray emissivity cube (usually derived from MHD) is a background subtracted 2-D map of the foreground SWCX emission with a per-pixel noise appropriate to the source, background, pixel size and integration time.

As described previously, at the start of Sect. 2, in Fig. 1 we presented slices in the XZ plane at Y = 0 of the MHD-derived SWCX X-ray emissivity, plus an example simulated two-dimensional image of count-rates as would be measured by SXI when SMILE is at a high altitude position, with the field of view has been directed towards the dayside magnetopause. The count rate image has been smoothed using a boxcar average, with a width of 5 pixels.

SXI simulated counts images are used to estimate the subsolar magnetopause position, using one or more of the methods determined by the SMILE Modelling Working Group (summarised in Wang and Sun 2022). One of these methods assumes an empirical shape of the magnetopause, that is then described by a limited set of parameters of the subsolar position and flaring exponent ($\alpha _{y}$) in both azimuth and elevation. The magnetopause can then be described as a distance (${\mathrm{r_{y}}}$) in the y direction (or similarly in the z, with an independent flaring exponent), in geocentric solar magnetic coordinates, where the positive X-axis points towards the Sun, the Y-axis is perpendicular to the Earth’s magnetic dipole and is positive towards dusk, and Z completes the orthogonal set. We use the following equation from Jorgensen et al. (2019), which is a reproduction of the Shue et al. (1997) result, where ${\mathrm{R_{MP_{0}}}}$ is the subsolar distance to the magnetopause, and $\theta $ is the angle from the x-axis:

1 $$ r_{y}(\theta ) = \mathrm{R_{MP_{0}} }\left ( \frac{2}{1 + \mathrm{cos\,}\theta}\right )^{\alpha _{\mathrm{y}}} $$

This can then be used to describe the three dimensional magnetopause as a distance from Earth ($\mathrm{r}(\theta , \phi )$), where $\theta $ is the angle from the X-axis and $\phi $ is the rotation about the X-axis starting from the Y-axis in the right-hand direction:

2 $$ r(\theta , \phi ) = \frac{r_{y}(\theta )\, r_{z}(\theta )}{\sqrt{[r_{z}(\theta )\, \mathrm{cos\,}\phi ]^{2} + [\mathrm{r}_{\mathrm{y}}(\theta ) \,\mathrm{sin\,}\phi ]^{2}}} $$

The method to derive boundary information from the simulated SXI data is as follows. We use the 3-D X-ray emissivity cube described in Sect. 2 to derive a predicted (model) SXI map of the exospheric SWCX emission by running the cube through the simulator but, in this case, without the application of noise or background subtraction. This model map can be compared to the data (derived from the MHD simulations) via a $\chi ^{2}$ calculation and a $\chi ^{2}$ minimisation routine used to find the empirical model parameters which best fit the data.

The resulting parameters of interest in this paper are the subsolar magnetopause position, and the flaring in the y-direction. The simulations were run twice. Firstly, no prior knowledge of the magnetopause shape along the flanks was assumed. This was the SXI ‘free’ run. Secondly, using the results of Sect. 2.2, $\alpha _{y}$ was constrained at a value of 0.25, which was set as it is the approximate average of the values derived from the OCB field-line tracing to the flank magnetopause fitting. This was the SXI ‘fixed’ run.

### Comparison of OCB-Tracing and SXI-Fitting at Dusk-Side Magnetopause Flank

**Fig. 6:**
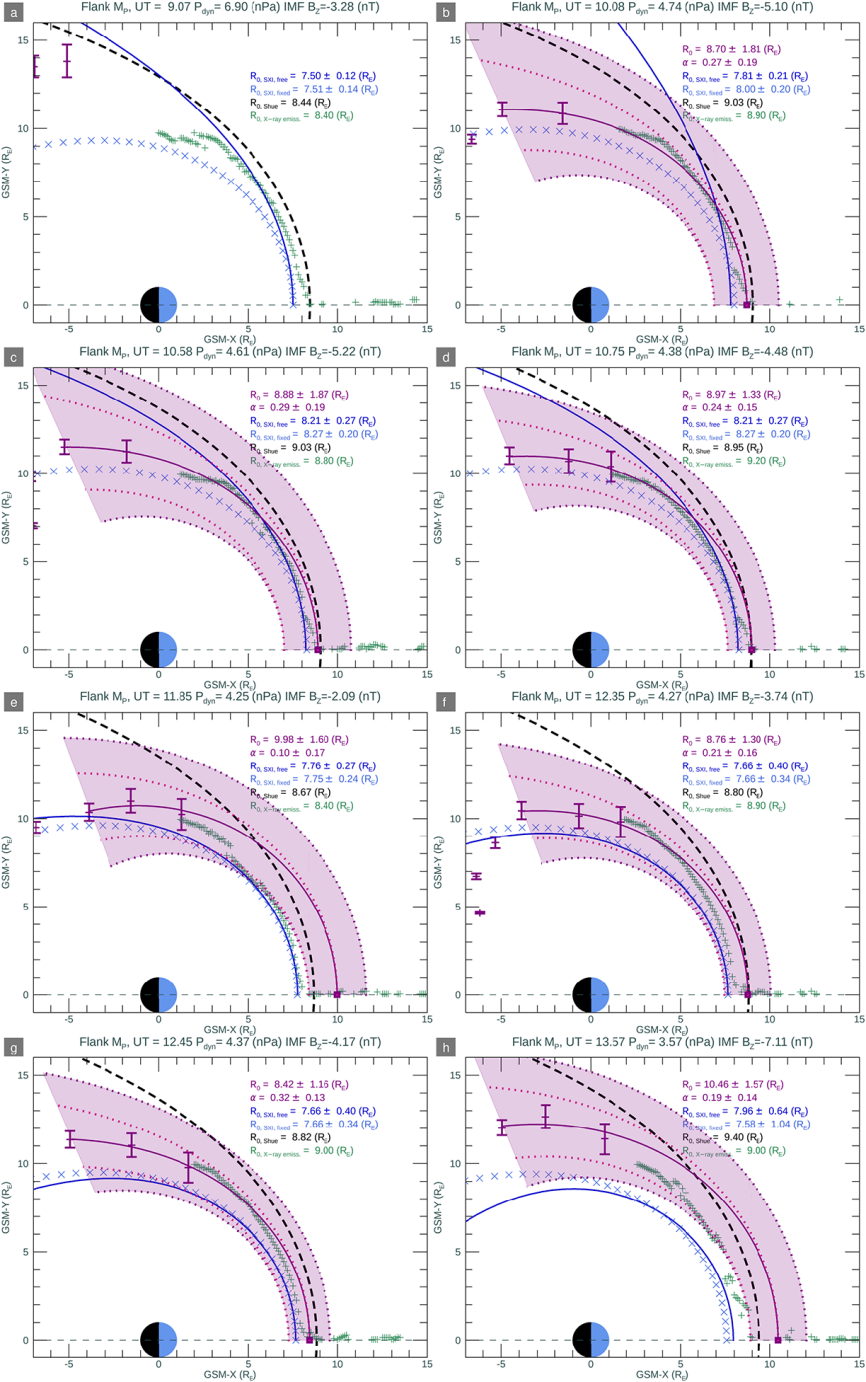
Flank Mp positions at each time step; Sun to the right. Purple points with error bars mark flank positions traced from the ionosphere, with the model magnetopause fitted to these points as a solid purple line. A purple square marks the subsolar magnetopause position. The light purple shaded area bounded by purple dotted lines is the estimated error on the magnetopause location given the OCB-derived fit. Pink dotted lines mark the flank magnetopause, if taking the lower/upper limits of the magnetopause stand-off position using the minimum/maximum flaring exponent. The Shue and Song (2002) empirical magnetopause is shown as grey long-dashed line. Green crosses mark the inner Y-boundary of the X-ray emissivity cubes at a given X-position. The SXI-simulator derived magnetopause positions with either the dark blue line (free, unconstrained fits) or the light blue ‘x’ symbols (fixed $\alpha $ in the y-direction), are described in Sect. 2.3

In Fig. 6 we plot a grid of flank field-line traced positions. In each panel, we plot the OCB-traced projections in the GSM Y-direction to the dusk flank magnetopause, in black, with estimated error bars. In each plot, the purple line traces the model magnetopause fit to these points. To estimate the subsolar location of the magnetopause, we use IDL procedure MPFITFUN from the Markwardt library (see Declarations) to fit the function of Eqn. (1) to the flank positions. Points that contributed to the magnetopause fitting were taken from equatorial distances in the X-direction greater than −5.0 $\mathrm{R_{E}}$, and those originated from ionospheric positions with MLTs of less than 20 hr. From this fitting we infer the subsolar distance to the magnetopause, $\mathrm{R_{MP_{0}}}$. The fitting function requires symmetrical errors in y, so we assume the largest absolute error in the y-direction at each x-position to use in the fitting function. The estimated flaring exponent and subsolar magnetopause distances are listed in Table 1, as well as the Shue and Song (2002) model. In the figure, the shaded purple area bounded by the purple dotted lines mark the inner and outer extremes of the magnetopause shape, given the estimated errors on either the stand-off distance or the flaring exponent derived from the auroral-emission field-line traced data, as noted in the purple text in the panel annotations. The inner and outer extremes were calculated using the form of the magnetopause in Equations (1) and (2). Pink dotted lines mark either the magnetopause shape, as calculated using the lower extreme of the magnetopause stand-off distance with the maximum $\alpha $ flaring exponent, or with the fitted magnetopause stand-off-distance with the maximum $\alpha $ flaring exponent. An empirical Shue and Song (2002) model magnetopause is shown in the black dashed line. We also plot the inner boundary in Y of the X-ray emissivity cubes at a given X-direction, as shown by the light green crosses, plus the SXI-simulator derived magnetopause positions with either the dark blue line (free, unconstrained fits) or the light blue ‘x’ symbols (fixed $\alpha $ in the y-direction), which are described later in Sect. 2.3.

### Summary of OCB Tracing and SXI Fitting

**Fig. 7:**
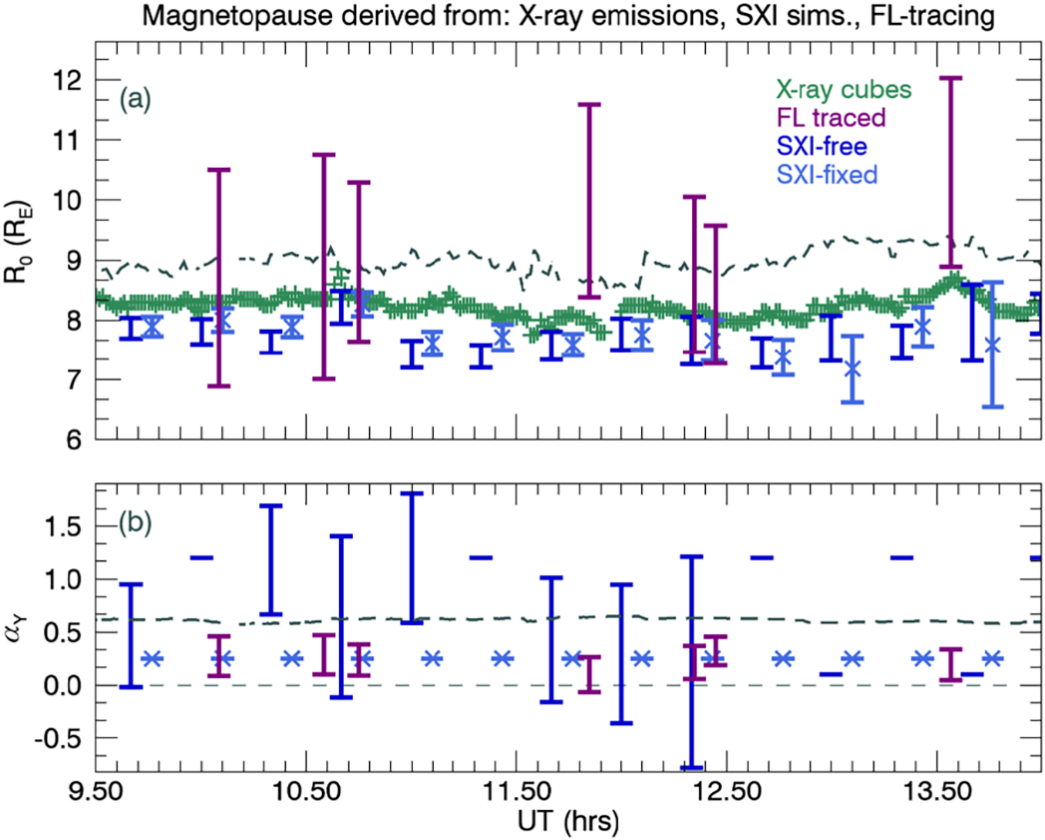
Panel (a) The magnetopause stand-off distance by time, and in Panel (b) the flaring exponent $\alpha $, In Panel (a) we also plot the empirical model of Shue and Song (2002) (grey, dashed line), and the stand-off distance from the X-ray emissivity cubes (green crosses). In Panel (b) a thin dashed line at zero is plotted to guide the eye, plus a thicker dashed line to show the Shue and Song (2002) value of $\alpha $. In both panels, we plot the field-line traced observations of the polar cap (purple, with errors), or as extracted by the SXI simulator with either free (dark blue) with associated errors where constrained, or fixed (light blue, ‘x’ symbol) parameters

We summarise the results from the field-line tracing and the SXI simulator results as a time series in Fig. 7, for the magnetopause standoff distance (panel a) and flaring exponent $\alpha $ (panel b). Panel a compares the magnetopause as obtained from the model of Shue and Song (2002) (grey dashed line), from the X-ray emissivity cubes (green crosses) as defined as the inner boundary of the magnetosheath emission in the subsolar direction, and as derived from the auroral emissions data (purple, with vertical error bars). In both panels the SXI-simulator derived results are plotted from either the free (dark blue), or fixed (light blue, ‘x’ symbol), with error bars. In panel (b) we plot the flaring exponent as determined by the OCB-traced data or from the SXI-simulator (with this to a fixed value for the ‘fixed’ case). The free parameters without vertical error bars indicate a badly constrained fit. An estimate of the Shue and Song (2002) derived flaring exponent, $\alpha $, is shown as the thick grey dashed line. In addition, we have added an estimate of the location of the OCB to Fig. 3, as derived from the fixed SXI results as field-line traced inwards from the flank magnetopause, back along field lines to the ionosphere, to a particular co-latitude which is extended as a circle, as shown by the green-dashed line.

## Discussion

For the 28 May 2010 case, we have presented solar wind, IMF, and auroral emissions data during a mild and isolated substorm, along with simulated X-ray emissions data and the results of observing the upstream magnetosphere using SXI. Note that in many cases dayside and nightside magnetic reconnection processes will vary independently, competing to change the open or closed flux content of the polar caps and leading to a more nuanced and complicated picture. Using an isolated substorm allowed us to assume dayside magnetic reconnection as the dominant process in driving changes in the coupled system.

We have compared the magnetopause subsolar position and inferred magnetopause shape as derived from SXI data, firstly without any prior constraints on the magnetopause position, and then later, with constraints imposed via auroral emissions data, as a proxy for UVI or other auroral emissions observations, obtained at the high-latitude ionosphere. The observational data allows for an estimation of the OCB to be traced to the afternoon flank of the magnetopause, from which a subsolar magnetopause distance is derived, along with an estimation of magnetopause flaring along that flank. SXI constrains the subsolar magnetopause position quite well independent of the constraints implied from the ionospheric data, although a systematic Earthward offset from that predicted by Shue and Song (2002), and from the estimated magnetopause position from the SWCX emissivity (MHD-based) cube data is observed, as shown in Fig. 7, panel a. The magnetopause stand off distance estimated from the SWCX X-ray emissivity (MHD-derived) cubes only gives stand off distances marginally closer to the Shue and Song (2002) empirical model as compared to the magnetopause stand off distance derived from the SXI simulator. In practice, during the operational phase of SMILE, we will seek verification and validation of magnetopause crossings from other available spacecraft to compare with the results from SXI. Note that the SXI results here are MHD-model dependent and dependent on the functional form as given in Equation (1) and Equation (2). Other magnetopause forms are currently being investigated by the SMILE Modelling Working Group as part of the preparations for the mission, e.g. Wharton et al. (2025), and are likely to be embedded in the SXI simulator nearer launch. The comparison with the OCB field-line traced magnetopause, obtained from observational data of the ionosphere, is magnetic-model dependent, which is itself based on a limited set of parameters. Both the OCB, field-line traced result and that from the free-exponent SXI fitting give a flaring shape along the flanks that is mostly Earthward of the Shue and Song (2002) implied shape, as shown in all but the first two panels of Fig. 6. In Fig. 7, panel b, the SXI-free flaring exponent shows considerable variance, albeit with large errors, as compared to the flaring exponent derived from the auroral emissions data (purple bars). There is little variation in the nearly flat empirical Shue and Song (2002) flaring exponent. The SXI-derived OCB, using the fixed flaring exponent and projected from the flanks inwards to the ionosphere, does not match the dayside auroral emission at all well, as shown by the green-dashed line in Fig. 3. The implication is that the SXI struggles to constrain the shape of the magnetopause at large azimuth away from the subsolar direction. The limits of SXI performance can only be determined with real observational data after launch, when the dependence on variable solar wind flux and other parameters will be investigated.

The observational auroral emission data we have used in this paper originates from the SSUSI instruments on board the DMSP satellites. The cadence of measurements from SSUSI makes this a poor proxy for the UVI instrument, but it is the best medium to large-scale auroral imager that is currently available and it allows us to image various aurora phenomena in sectors of the polar cap. UVI will surpass SSUSI in terms of both temporal and spatial coverage, as it will provide grey scale images of the whole Northern Hemisphere polar cap at least 1 minute resolution, comparable to the performance of previous ultraviolet imagers that flew over two decades ago. We have used the UVI-proxy data to constrain the flaring of the dusk side magnetopause in this paper, and feed this information back into the SXI routines to increase the performance of the SXI simulator. When UVI is operational, we will be able to extend this approach to both magnetopause flanks, and explore questions such as magnetopause asymmetry. To obtain this constraint we have employed field-line tracing techniques. This is very sensitive to the magnetic latitude of the OCB, whether this is determined from the auroral emissions or other data, such as FACs in this work. Care had to be taken to avoid medium and small scale features such as auroral arcs and cusp spots when determining the OCB from the auroral emission data, and the UVI will be no exception. It is worth noting that instead of absolutes of the position of the OCB, the change in the open magnetic flux content is the more important indicator of solar wind-magnetosphere-ionosphere coupling dynamics, as is the time history of the system.

The field-line tracing is also wholly dependent on the empirical model of the magnetosphere that is being used, instead of depending only on solar wind dynamic pressure, the clock angle of the IMF, and an estimation of the ring current, for a particular date and time. Note that the current empirical models are not parameterised by open magnetic flux content of the polar cap. The error on the magnetic field strength of the T96 model has been found to have a mean of approximately 14% during quiet, non geomagnetic storm times (Song and Min 2022), although the errors specifically in the flank regions are unknown. The errors on the SXI magnetopause position in the flanks however, are large and likely dominate the uncertainties in the trace to the ionosphere (purple shaded areas in Fig. 6). The field-line tracing technique is in common use in magnetospheric-ionospheric coupling studies, and therefore a understanding of the limitations and error margins on this technique will continue to be important in the SMILE era. SMILE science will work to build a nuanced empirical model of the magnetopause, using the results of SXI with the open-closed field line boundary as estimated by UVI and ground-based auroral imagers. Note, other experiments may prove useful in estimating the OCB at different spatial scales and temporal cadences to UVI, and reveal phenomena that are otherwise unavailable to SMILE. These experiments may include the SMILE All Sky Imager network, that is distributed across several hours of magnetic local time in the North American sector taking auroral images at multiple wavebands offering insights into the energies of precipitating particles and spatially precise measurements of boundaries, for example, using red line emission (Gallardo-Lacourt et al. 2022). The OCB may also be approximated from the lower-latitude extent of the ionospheric convection pattern as determined by SuperDARN and AMPERE (e.g. Fogg et al. 2020) or SuperDARN and IMAGE (e.g. Imber et al. 2013). There is an offset between the SuperDARN-determined boundary and that of the return flow boundary between the region 1 and region 2 FACs, as determined by AMPERE (Fogg et al. 2020; Walach et al. 2025). Additional discrepancies between these boundaries are reported by Walach et al. (2025), however, during highly driven geomagnetic storm time conditions, possibly attributable to the different latencies between FACs and convection across the different phases of storm expansion and recovery. In addition, ground-based or other spacecraft estimations of the OCB will be able to fill in data gaps from SMILE, for example, during the perigee passage of the spacecraft when the imagers are switched off, aiding long-baseline time series analyses. The work of the Ground-Based and Additional Science Working Group and wider solar-terrestrial community will make significant contributions to not only enhancing the results of SMILE, but also offering distinct measurements of the global system from an alternative perspective.

In Figs. 3 and 4 we also include a circle at all MLTs but offset from the magnetic pole by 5 degrees antisunward and 2 degrees duskward, (Milan et al. 2009) that indicates an SXI-derived OCB, as shown by the dark green dashed lines. This is estimated by field-line tracing from the dusk flank magnetopause, as determined from the SXI-derived magnetopause position using the fixed flaring exponent, inwards to the ionosphere to a particular co-latitude. A circular shape, offset from centre, is assumed from this co-latitude, which may not be representative of the open field-line region shape, especially in the dayside sector, but it is used here as a first approximation. In the dayside polar cap, phenomena such as bending arcs (also known as large-scale poleward moving auroral forms Carter et al. (2015)), cusp spots (e.g. Zhou et al. 2024), or transpolar arcs (reviewed in Hosokawa et al. 2020), and as described in Sect. 1, may be found, making the shape of the open polar cap region asymmetrical.

In this work we have used a fixed integration time of 20 minutes to analyse the SXI data. However, in reality, optimised or customised integrations will be explored by a user of the data, depending on the science question under scrutiny and the strength of the X-ray signal, itself dependent on the incoming solar wind conditions. The primary product that is provided by the SXI team is a list of background removed X-ray photons that can be subsequently selected to create secondary products such as images, spectra, or time series. Therefore, a user will be able to minimise the temporal resolution of the derived magnetopause position and shape, whilst ensuring good signal to noise. Archival studies will allow alternative partitions of data, for example, based on similar contemporaneous interplanetary conditions, although we have not explored those possibilities here.

**Fig. 8:**
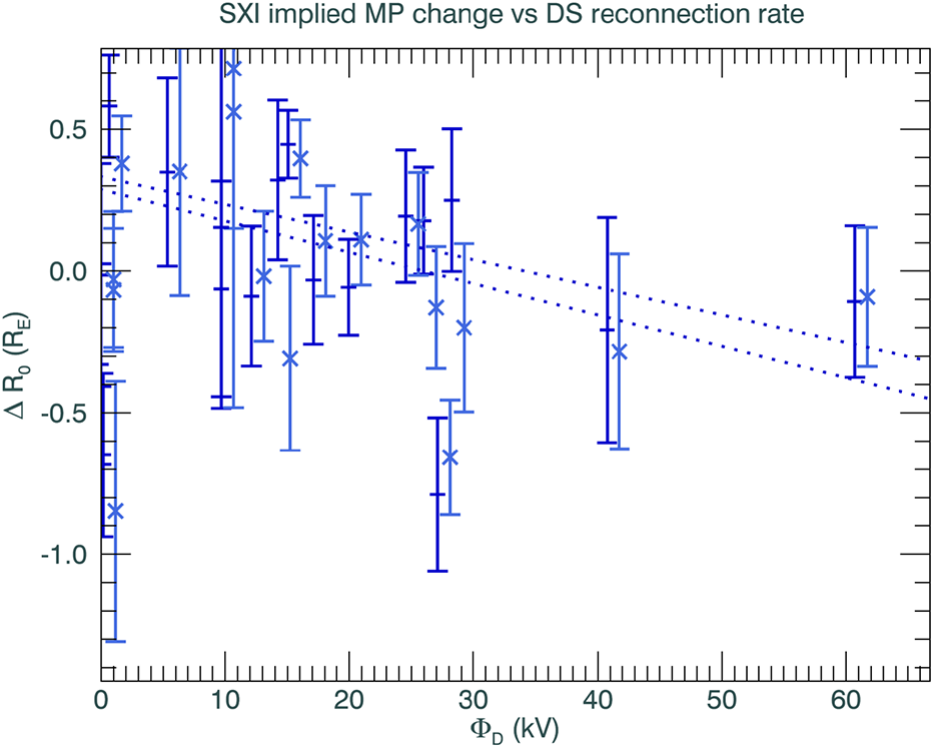
The change in the SXI-derived magnetic stand-off distance versus the dayside magnetic reconnection rate, for the free-parameter (dark blue crosses) or fixed (light blue, ‘x’ symbol) SXI-simulator fits. The fixed parameter fits have been offset slightly in the x-axis direction by 1 kV to aid visualisation

In Fig. 8 we plot the change in the SXI-derived magnetopause stand-off positions, for both free and fixed flaring exponents) versus the dayside magnetic reconnection rate, between 09:30 and 14:00 UT. The dayside magnetic reconnection rate is calculated from the change in magnetic flux with time, using the empirical relationship between rate and interplanetary magnetic field from Milan et al. (2012). We plot both the SXI fits using SXI-fitting with parameters fitted freely (dark blue crosses) or by fixing the flaring exponent (light blue ‘x’ symbols). We also include a linear regression to both the free and fixed-SXI fits, that discounts the lowest dayside reconnection rates below 5 kV. The fits are shown in the figure using dark and light-blue dotted lines. Both linear relationships, although with shallow gradients, show that, as the dayside magnetic reconnection rate increases, the SXI-found magnetopause stand-off distance moves inwards (negative), as expected. There is considerable scatter in the change in the stand-off distance found. There is also a suggestion of the magnetopause moving sunwards at very low dayside reconnection rates, which may be linked to barely southward clock-angles or northward turnings, or errors in the propagation of the OMNI data set that has been propagated from upstream monitors to the bow shock. Such sunwards movements of the magnetopause at low dayside reconnection rates may be explored during the SMILE era. We have yet to explore other competing factors, for example, internal to the magnetosphere, that may moderate magnetopause motion, however, this work provides an illustration of how relationships between dayside driving and SXI-derived magnetopause motion may be explored to satisfy one of the major research goals of the SMILE mission in understanding dayside magnetic reconnection.

## Public Engagement with the SMILE Mission

The novel images that SMILE will provide, and the mission as a whole, offers a fantastic opportunity for engaging with the public. This naturally links with the science, aims, and previous experiences of many people in the solar-terrestrial community, facilitating the links to engagement programmes with considerable involvement from members of the GBAS working group. The separately run, but with considerable cross-over, SMILE public engagement (PE) working group reports to the mission Science Working Team about various activities using SMILE directly or indirectly with regards to engagement and outreach. Outreach is considered passive, such as memorabilia associated with the mission or certain talks and media appearances. Engagement, on the other hand, requires a two-way relationship with the public to be established, and may involve activities such as data analysis by both scientists and the general public, working and discussing the results together. The group has so far led workshops and stands for various community groups, university open days, and science fairs, including the British Science Festival and Royal Society Summer Exhibition, as well as invited public talks and talks to more specialist interest groups.

Before the COVID pandemic, it was hoped that long term relationships could be built up with school children or community groups in the Leicester area, local to the SXI team, so that a cohort of students could follow the trials and tribulations of the mission as it progressed through design, build, testing, and operations, using the mission as an example of team working, international collaboration, resilience, and problem solving. Relationships with schools and community groups were established, but, since the pandemic, the nature of the engagement with these groups has somewhat changed. Members of the working group engaged with students around 14 years old as part of the well-established and UK-wide Orbyts project (Dunn et al. 2018), using real science data to explore the solar wind charge exchange process which results in the X-ray signal that will ultimately detected by SXI. Orbyts projects aim to result in tangible scientific outputs in student-led, peer-reviewed publications. Other members have interacted on a longer-term basis both with a mixed-age community group in a socio-economically deprived area of Leicester and for a summer school for disadvantaged pupils in London, using SMILE and SMILE-related science as the basis of several workshops. SMILE has been used as a backdrop for talks to various age groups regarding careers in Science, Technology, Engineering, and Mathematics, in various countries of the SMILE consortium.

SMILE will launch close to solar maximum, and there is increasing public awareness of the potential effects of space weather, conversation of which has entered into mainstream media. For example, the recent extreme solar storm of 10-12 May 2024 was reported on news channels worldwide, resulting in many sightings of the aurora at low latitudes for people that are normally unaware of the phenomena. This gives SMILE a current hook to engage with those people that might normally have little or no experience of space physics.

The focus has so far been on engagement, so that as much as possible the public is an active participant in any event. There is a myriad of examples of citizen science, particularly in the astronomy community, and SMILE will do well to build on these examples. One such ongoing activity is the Maryland Space Weather UnderGround build and test an array of cost-efficient, research-capable magnetometers across the United States for supporting the Sun – Solar Wind – Earth interaction research (Connor and Keesee 2021). This links up naturally with endeavours by the wider GBAS community involved in other solar-terrestrial missions, such as NASA’s Electrojet Zeeman Imaging Explorer, whose team run a similar shoe-box sized magnetometer kit for deployment in schools. These large programmes offer a plausible and relevant link to SMILE science and a coordinated engagement project with these existing programmes would increase the impact and reach of all the contributing missions, including SMILE.

The working group also has endeavoured to engage with groups that are under-represented or traditionally not embedded or invested in science engagement programmes. For example, this includes very young children, through the aurora-related ‘Aurora and Spotty’ book, and the visually-impaired and neuro-diverse community through the Tactile Space project https://tactilespace.le.ac.uk that was inspired by the astronomy-focused Tactile Universe (Bonne et al. 2018). Engagement activities are also supported via outreach materials as provided by ESA, anticipated physical SMILE paraphernalia, and social media posts and web pages.

There is still great potential for the mission as a whole to be seen as a springboard for SMILE-related science (e.g. geomagnetism, stellar winds, electromagnetic spectrum), or for more periphery and tangential studies, for example, as inspiration for art, languages, or international relationships. One major benefit of SMILE is its ability to provide a visual representation of the rather esoteric science of solar terrestrial space plasma interactions, which is of interest to the broader community to utilise for their own science outreach and engagement programmes. This has been fruitful for missions such as Swarm, for example, when linking up with ground-based auroral observations made by citizen scientists (Nanjo et al. 2024). The SMILE PE working group will continue to explore engagement opportunities and those for outreach, including immersive experiences such as using music, or Virtual and Augmented reality, to bring SMILE science to a wider audience as possible.

## Conclusion

The power of SMILE is its multiple perspectives on the Sun-Earth system, and the ‘L’ in the acronym, ‘Link’, is key to the mission philosophy and future success. SMILE will radically alter our views on how the dynamics of the magnetosphere play out as we can finally observe cause and effect processes across the system. The observations and measurements made by SMILE’s instruments will not be considered in isolation, rather they will be presented in respect to other results from the wealth of experimentation probing the ionosphere and magnetosphere. Previously, in papers exploring the efforts of the SMILE Ground-based and Additional Science Working Group, we have explored how ground-based experiments and other spacecraft can be used to maximise the impacts of the SMILE mission, by describing how the system-scale observations from SMILE can be extended to multiple spatial and temporal scales and lead to a holistic understanding of magnetosphere-ionosphere coupling. These experiments offer different information to SMILE, e.g. a determination of the return flow boundary from patterns of FACs at the ionosphere. This paper, conversely, offers a preliminary examination of how SMILE’s X-ray camera viewing the magnetopause, and the Ultraviolet camera observing the Northern Hemisphere aurora, may work together in association with additional experimental data sets. These observations, e.g. of FACs or from other, non-SMILE auroral emission data, may improve the performance of the processes leading to the SXI-derived magnetopause position. Future work will continue to explore synergies between SMILE and ground-based and space-based data sets. SMILE will continue to inspire the general public, through well-structured engagement opportunities, and through experiences and outreach materials that move beyond the traditional obstacles to science involvement for various sectors of the community.
